# Transvenous salvage of coil migration with intraprocedural pulmonary circulation protection and successful transvenous coil embolization for the treatment of giant high-flow renal arteriovenous fistula

**DOI:** 10.1259/bjrcr.20180110

**Published:** 2019-01-07

**Authors:** Ka Yin Gregory Lee, Yee Tak Alta Lai, Kam Wing Warren Leung

**Affiliations:** Department of Radiology, Pamela Youde Nethersole Eastern Hospital, Hong Kong, China

## Abstract

Transcatheter embolization is a well-established treatment for renal arteriovenous fistula (AVF) in selected cases. Transarterial approach has been the conventional route of access of the AVF. In large arteriovenous shunts, however, transarterial approach inherits the risk of distal migration of embolization material with subsequent pulmonary embolism. We report a case of giant high-flow renal arteriovenous fistula treated with coil embolization. Arterial approach was attempted with double catheter technique, however complicated with coil mass dislodgement. We have retrieved the coil mass via transvenous route with simultaneous pulmonary circulatory protection and subsequent successful transvenous coil embolization with complete obliteration of the AVF was performed.

## Case

**Figure 1.**
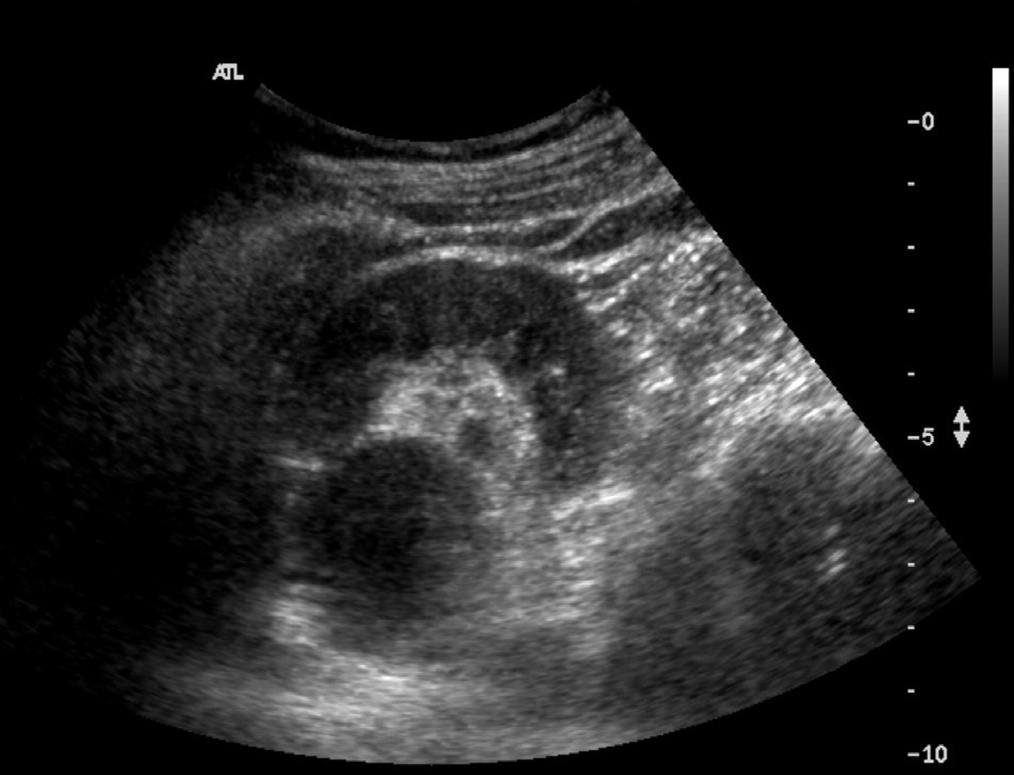
Ultrasound study of the left kidney during routine health check. Ultrasound of the left kidney revealed an extrarenal pelvis with suspected left hydronephrosis (arrow) and hydroureter.

**Figure 2.**
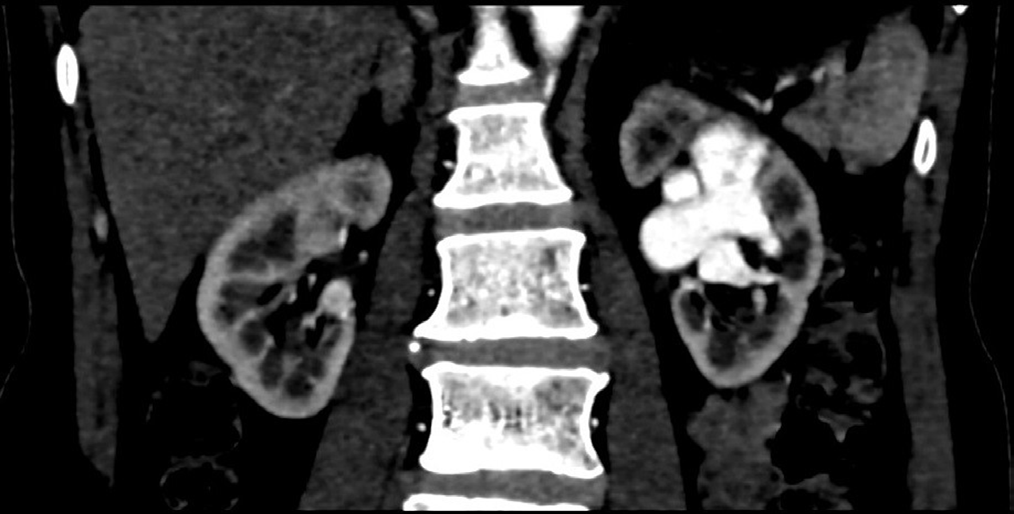
Contrast CT Urogram (arterial phase-coronal). At least two direct fistula sites seen from left renal artery leading to dilated left renal vein (arrows).

Our patient was a 54-year-old female with unremarkable past medical history. She was noted to have suspected left hydronephrosis and hydroureter on an ultrasound study during routine health check (Figure 1). Subsequent contrast CT urogram was arranged at the Department of Radiology, Pamela Youde Nethersole Eastern Hospital, which noted aneurysmal dilatation of the left renal artery and its segmental branches. Early opacification with dilatation of the left renal vein up to 20 mm at arterial phase was also seen (Figure 2). Imaging features were compatible with a left renal arteriovenous fistula (AVF).

The patient had a history of trauma during childhood, in which she fell on steel sticks with puncture wounds over her left back. She did not seek medical help at that time. In view of the significant size of the shunt and to prevent further complications that may arise, the patient preferred to undergo endovascular treatment after discussion of risk and benefits.

**Figure 3.**
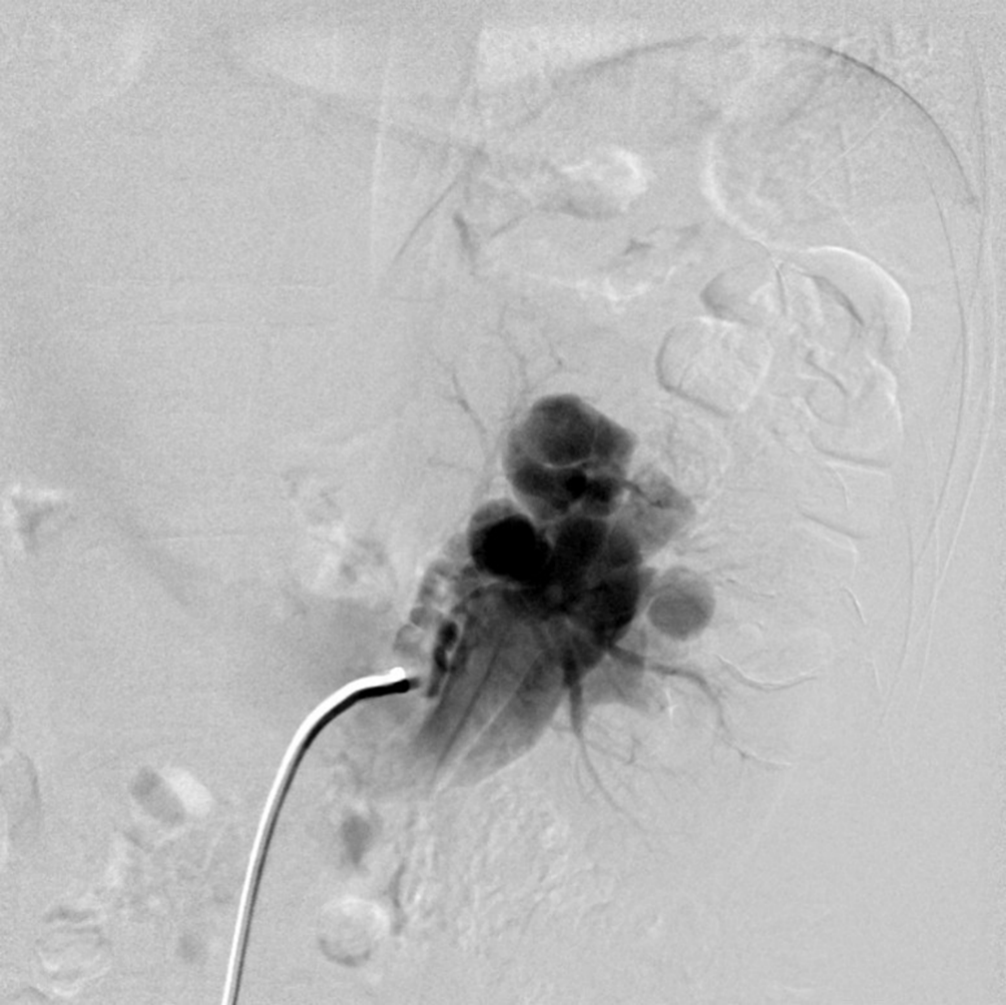
DSA pre-embolization. Catheter tip is situated at left renal artery (arrow). Dilated and tortuous left renal vein with early opacification (arrow head), which occur before left renal parenchymal staining due to abnormal shunting. DSA,digital subtraction angiogram.

Angiography and endovascular therapy were performed using the Siemens Axiom-Artis system (Siemens Medical Solutions, Erlangen, Germany). On the pre-procedural left renal artery digital subtraction angiogram (DSA), a large AVF was confirmed located at the left renal upper pole. At least two direct fistula sites were seen on real-time fluoroscopy leading to the early opacified dilated left renal vein, which measured about 24 mm in calibre. Bulbous dilatation of the vessel just proximal to the fistulas are seen. (Figure 3).

**Figure 4.**
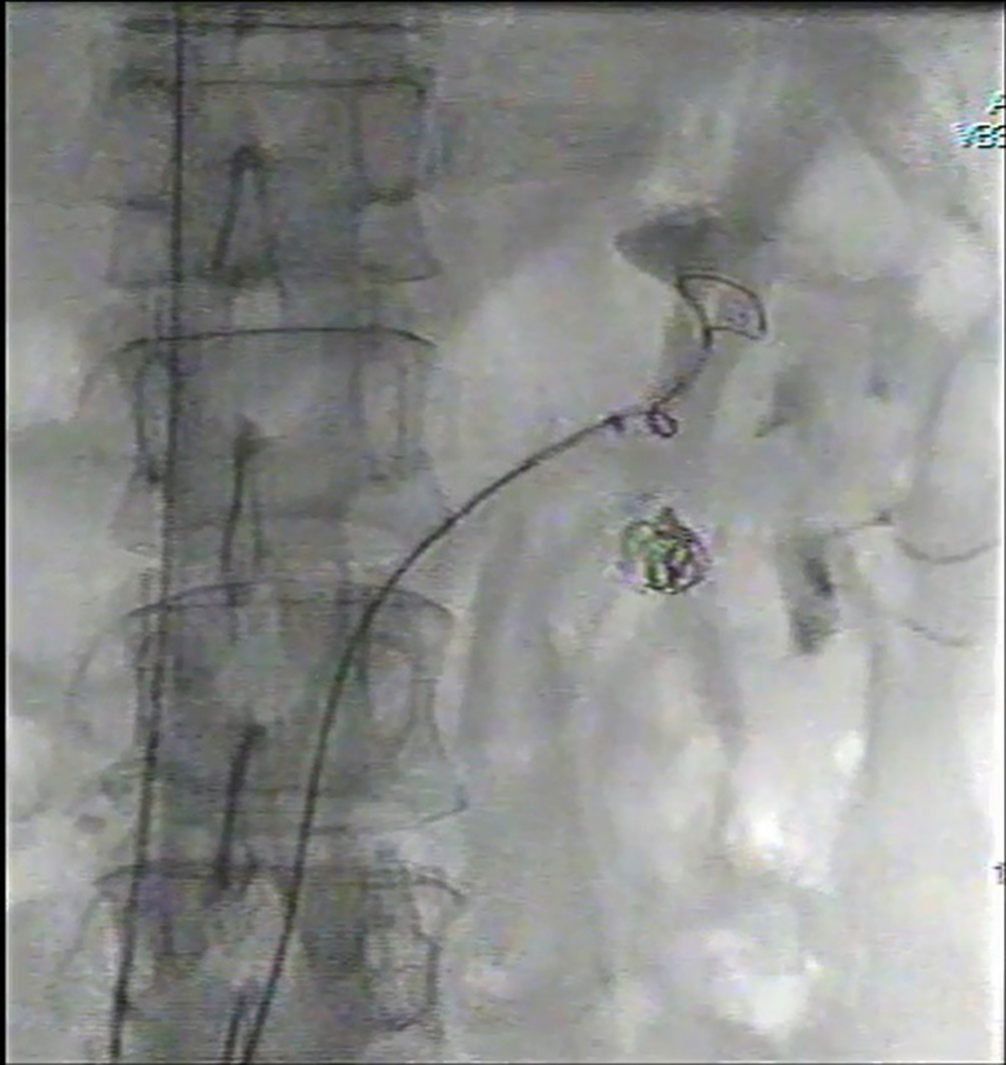
Captured image from intraprocedural cine. Coil mass (arrow) migrated distally from the left renal artery to the dilated left renal vein via the giant high flow renal AVF. AVF, arteriovenous fistula.

We have navigated into a distal left upper pole arterial branch, just adjacent to the site of fistula with the use of a 2.8 Fr Direxion HI-FLO microcatheter (Boston Scientific Corporation, Natwick, MA) and 0.014’’ Transend EX Floppy micro-guidewire (Stryker Neurovascular, Fremont, CA). We made two attempts in coiling via this route. With the use of a Ruby detachable coil (Penumbra, Alameda, CA, 8 mm × 60 cm), failure to anchor the vessel wall with repeated prolapse into the left vein was encountered. In our second attempt with the use of double-catheter technique, another 2.5 Fr Renegade microcatheter (Boston Scientific Corporation, Natick, MA) was navigated to the bulbous dilatation just proximal to the AVF. An Interlock-18 Fibered IDC Occlusion System (Boston Scientific Corporation, Natwick, MA, 10 mm × 30 cm) was introduced via the second microcatheter in attempt to entangle the Ruby coil; however, accidental deployment was encountered with the coil mass migration into the left renal vein distally (Figure 4).

**Figure 5.**
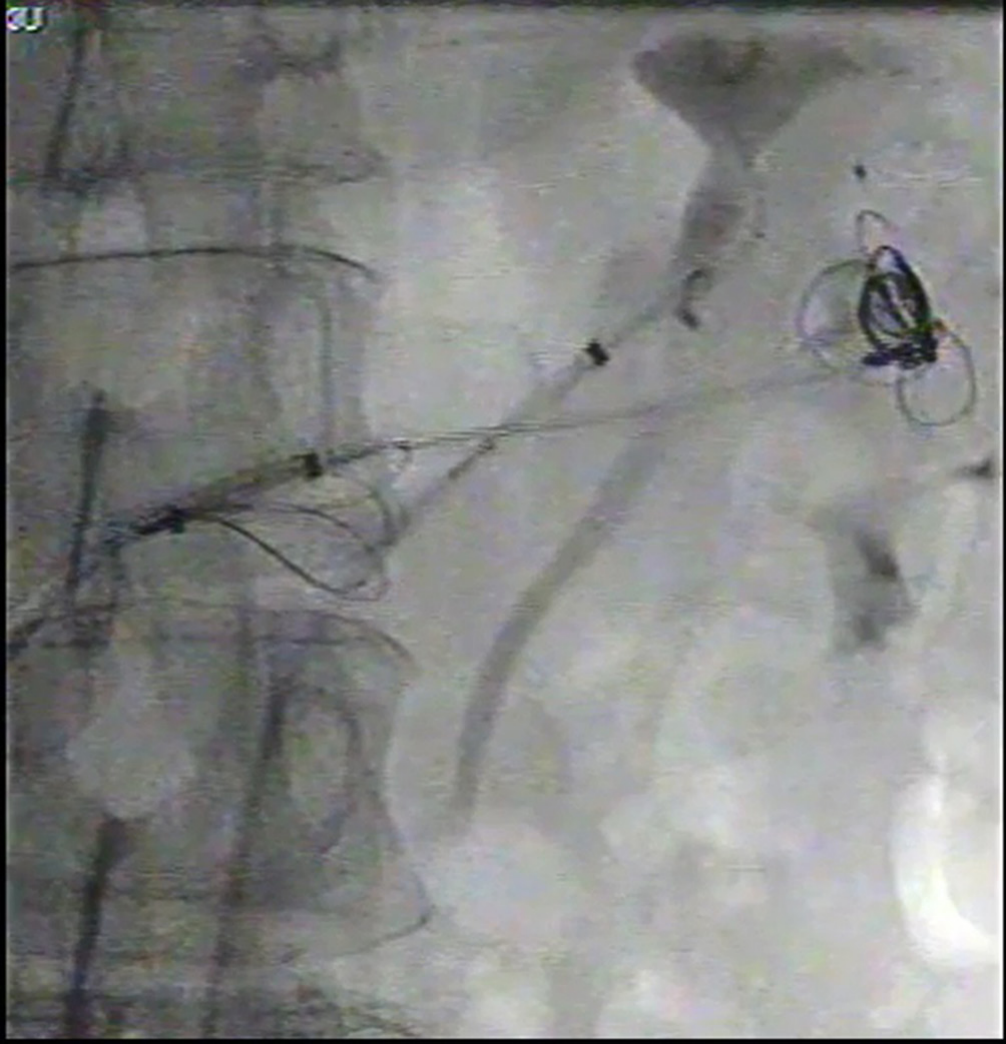
Captured image from intraprocedural cine. The first snare (arrow) was placed at the left renal vein to prevent migration of coil mass, while the second snare (arrow head) was used for coil mass retrieval.

**Figure 6.**
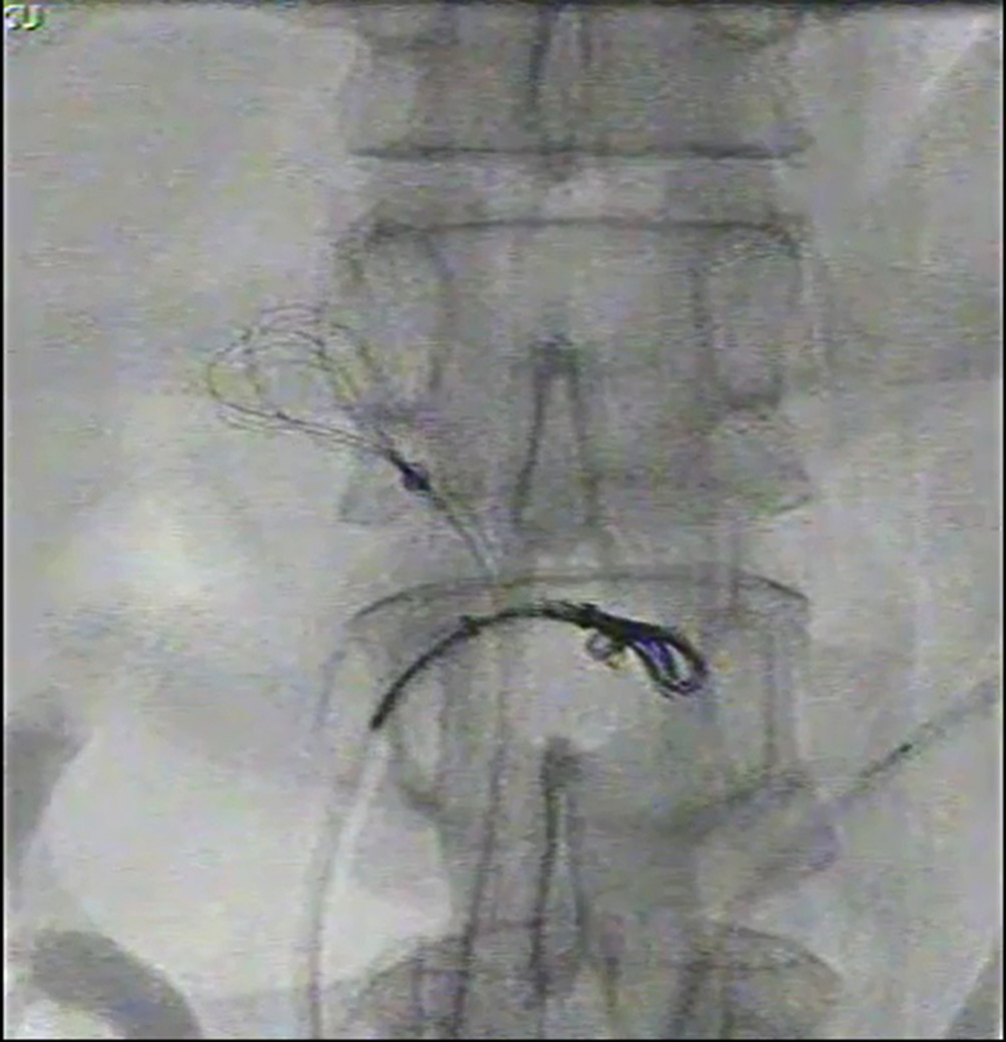
Captured image from intraprocedural cine. While the coil mass was retrieved towards the left groin by the second snare (arrow head), the first snare (arrow) was repositioned to the IVC for protection against coil mass migration into the pulmonary vasculature. IVC, inferior vena cava.

We have decided to snare and retrieve the coil mass through transvenous approach. Via bilateral common femoral vein (CFV) punctures, two 6 Fr guiding sheaths (Flexor Guiding Sheath, Cook Medical, Bloomington, IN) were inserted through each CFV with both tips placed at the left renal vein. Two 6 Fr EN Snare Endovascular Snare Systems (Merit Medical, South Jordan, UT) 12–20 mm were used. The first snare was placed at the left renal vein to prevent migration of coil mass, while the second snare was used for coil mass retrieval (Figure 5). While the coil mass was retrieved towards the left groin, the first snare was repositioned to the inferior vena cava (IVC) for protection against coil mass migration into the pulmonary vasculature (Figure 6).

After retrieval of the coil mass, we proceeded to access the renal AVF via transvenous approach. The wide calibre of the left renal vein resulted in challenging retrograde navigation and entrance into the fistula. Combined transvenous and transarterial approach and a through-and-through wire technique were utilized in effort to track the microcatheter from venous side-to arterial side. A 300 cm 0.014″ Synchro guidewire (Stryker Neurovascular, Fremont, CA) was threaded into the left renal vein from arterial side. The Synchro guidewire was then snared by a 4 Fr 10 mm Goose Neck snare (Medtronic, Dublin, Ireland) and then gently pulled back into the 6 Fr guiding sheath at the venous side. The attempt was unavailing as high resistance was encountered towards the end of pulling, probably due to acute angulation, thus this technique was abandoned.

**Figure 7.**
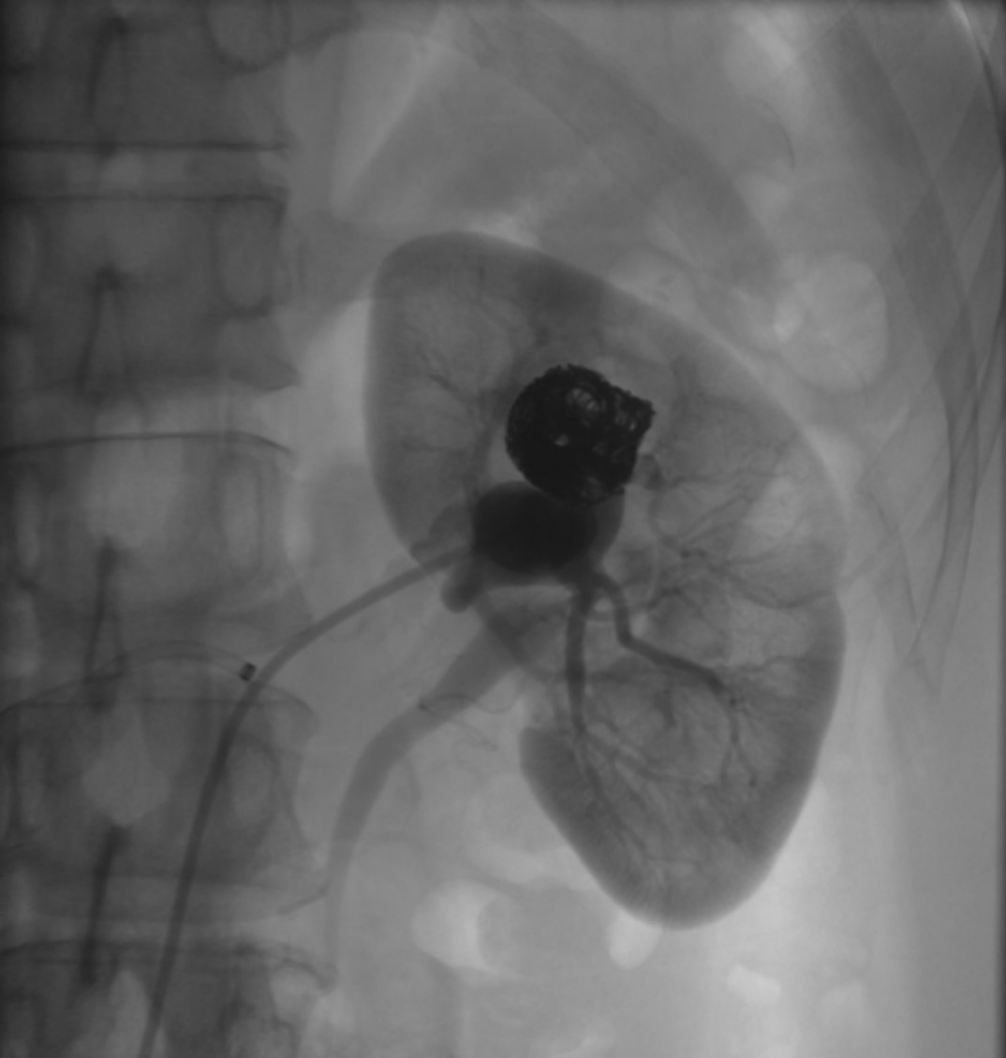
DSA post-embolization. Coil mass with successful obliteration of the left renal AVF. Normal renal parenchymal enhancement seen after successful embolization. AVF, arteriovenous fistula; DSA, digital subtraction angiogram.

The bulbous dilatation just proximal to the fistulas was finally successfully entered by transvenous approach, by establishing tri-axial access through venous side-using a 6 Fr guiding sheath at left renal vein, a 5 Fr Cobra one catheter (Cook Medical; Bloomington, IN) at left renal vein near the fistula, and a 2.8 Fr Direxion HI-FLO microcatheter at the bulbous dilatation. Retrograde navigation to the left upper pole renal arterial branch leading to the fistula was performed. This arterial branch and the bulbous dilatation were packed by six detachable coils, including three Ruby Standard coils (18 mm × 57 cm, 16 mm × 60 cm, 7 mm × 40 cm), one Ruby Soft coil (8 mm × 60 cm) and two Penumbra Occlusion Device (POD) Packing Coils (60 cm, 30 cm) (Penumbra, Alameda, CA). Complete obliteration of the fistula with preservation of most of the renal arterial branches was achieved on immediate post-treatment angiogram (Figure 7).

**Figure 8.**
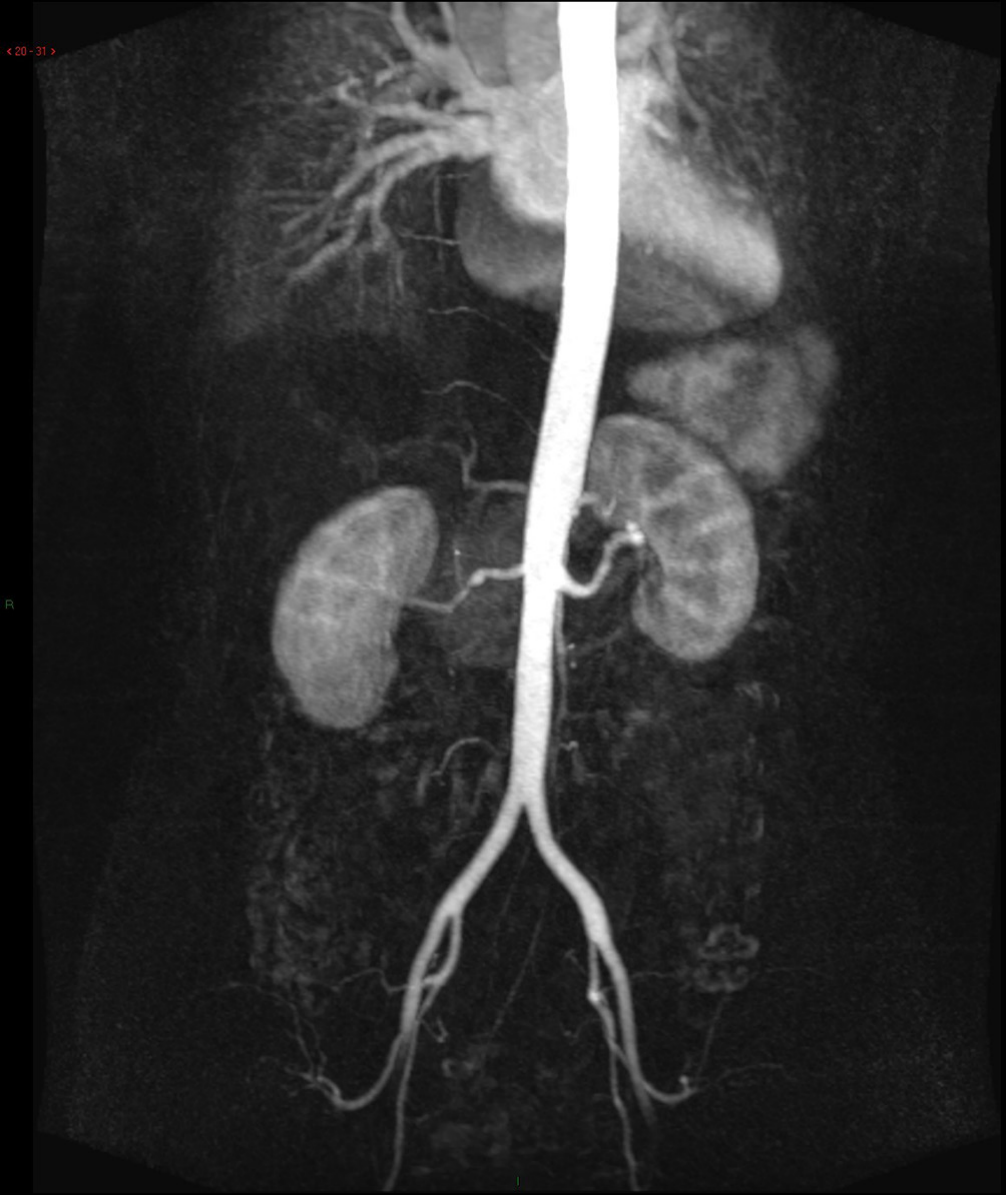
TWIST 4D MR angiography. Follow-up MRA in 3 months show no evidence of early venous shunting into the left renal vein, suggestive of successful obliteration of left renal AVF. AVF, arterio venous fistula.

On follow up time-resolved MR angiogram acquired with a 1.5-T scanner (Siemens Magnetom Avanto, Siemens AG, Erlangen, Germany) 3 months later, the left renal vein showed reduction in calibre and no early venous shunting, suggesting successful obliteration of the renal AVF (Figure 8).

## Discussion

Renal AVF is a direct communication between artery and vein without intervening vascular nidus. It is an uncommon disease entity with prevalence of about 0.04% in the general population.^1^ Majority of renal AVFs are acquired, which can be traumatic or non-traumatic in aetiology. Traumatic causes include blunt or penetrating trauma, biopsy and surgery. While non-traumatic causes include inflammation, neoplasm or renal arterial diseases such as dissection or fibromuscular dysplasia.^2^

Renal AVFs have a range of different presentations. They can be incidental with a bruit on physical examination, or can causes flank pain, haematuria, hypertension and high-output cardiac failure. No established guideline for treatment exists with accepted indications include large or progressive increase in size of the AVF, symptomatic patients with non-resolving haematuria or haemodynamic compromise from the AVF. Treatment options include endovascular approach and surgery.

In terms of their angio-architecture, traumatic renal AVFs are often seen as a single direct fistulous communication between renal artery and an adjacent vein with occasionally co-existing pseudoaneurysm. For non-traumatic cases, Cho et al has proposed a classification of peripheral arteriovenous malformation in terms of angio-architecture which others have adopted its use in renal AV shunts.^1,3^ This classification also serves as a guidance for choice of embolization material. In Type I non-traumatic AV shunts, a single or few arteries shunt to a single dilated draining vein. In Type II, multiple arterioles shunt to a single dilated draining vein. While Type III shunts are those with a complex vascular network formed between multiple arterioles and venules.

The goal of treatment for renal AVF is a definite closure of direct communication between the arterial and venous components. For traumatic renal AVF or Type I non-traumatic AV shunts, including our index case, the shunt should be occluded as closely as possible to the fistulous point to prevent renal infarction. Embolization with coils is the mainstay of treatment by encouraging stasis of flow and inducing thrombosis. Detachable coils (such as Ruby coils used in our case) are preferred to pushable coils to ensure safe sizing and accurate positioning before final release and deployment. Other described methods include the use of Amplatzer device, microvascular plug or stent-assisted coiling.^4^ N-Butyl cyanoacrylate (NBCA) use after placement of some coils have also been used to further achieve complete fistula occlusion.^1^ Particles such as Gelatin sponge or polyvinyl alcohol (PVA), and liquid embolic agents such as NBCA or Onyx maybe more suitable to be used in Type III arteriovenous shunts.^5^

In terms of the vascular access to the AVF, transarterial approach has long been used. However, distal coil migration is a well-known complication with reported incidence up to 8%.^6^ Such risk might even be higher while tackling a giant high-flow AVF like in our index case. Flow control with a double catheter technique was described by Maruno et al^1^ which might have a lower risk of distal migration of embolic material. Two guiding catheters were introduced to the renal artery via bilateral femoral approach, one of it being a 5–7 Fr Balloon catheter for occlusion of the feeding artery. Two microcatheters are then introduced to the fistulous segment via the guiding catheter and the balloon catheter respectively for application of embolic materials such as coils and NBCA. With flow control by the balloon catheter causing decrease in arterial flow within the fistula, risk of distal coil migration before establishing an adequate coil mesh might be reduced theoretically.

Simultaneous transarterial and transvenous coil embolization of large renal arteriovenous fistula was described by Nakayama et al.^6^ From the arterial side, a 5 Fr balloon catheter was introduced, with the similar aim of flow control as previously described. Both the arterial and venous accessed microcatheters were placed at the venous sac. From the venous microcatheter, embolization of part of the venous sac and draining vein was performed first, followed by embolization of the remaining venous sac and feeding artery via the arterial microcatheter. Theoretical benefit of first performing an incomplete embolization via the venous side—is that the pressure within the AVF would not increase, hence might reduce the risk of AVF rupture.

Transvenous embolization of renal AVF has not been discussed in the English radiology literature. In our patient, we have successfully gained transvenous access of the AVF with tri-axial access: a 6 Fr guiding sheath at left renal vein, a 5 Fr Cobra one catheter at left renal vein near the fistula and a 2.8 Fr Direxion HI-FLO microcatheter at the bulbous dilatation. Retrograde navigation to the left upper pole renal arterial branch leading to the fistula was obtained via Fathom-16 Guidewire (Boston Scientific Corporation, Natwick, MA) and Silverspeed 0.014 Hydrophilic Guidewire (Medtronic, Dublin, Ireland). This arterial branch and the bulbous dilatation were packed by six detachable coils (three Ruby Standard coils, one Ruby Soft coil and two POD Packing coils).

Transvenous access offers a benefit of allowing direct pulmonary circulation protection from migrated embolic material in giant AVF treatment. In our patient, transvenous approach not only allowed us to retrieve the coil mass which we have accidentally deployed, but also to place an additional snare in the proximal portion of left renal vein close to the IVC before the manipulation and retrieval of the coil mass in the distal renal vein, as a precautionary measure to prevent further inadvertent distal migration of the coil mass into the pulmonary arterial circulation. We subsequently repositioned the protective snare to the IVC when the coil mass was drawn through the IVC, common and external iliac veins, common femoral vein and the femoral sheath, and eventually removed via the left groin.

During the procedure, different pulmonary circulation protection strategies that could be offered were considered. Apart from the use of snare as previously described, a retrievable IVC filter was another option. However, there are other procedural complications from IVC filter deployment and retrieval which may occur, including malposition, defective filter deployment and vascular injury.^7,8^ In addition, even if the coil mass was trapped by the IVC filter, further measures would then be necessary to remove the filter and the coil mass, such as by means of a snare. As pulmonary circulation protection was desired only during the intraprocedural period, the use of a snare would be a safer and more cost-effective option.

In terms of approach to the left renal vein for coil mass retrieval in our patient, we have considered the options of right transjugular versus transfemoral approach. Benefits of a right transjugular approach include a potentially more favourable cannulation angle of the left renal vein from the IVC and obviation of additional femoral punctures. However, as time was of the essence for the coil mass retrieval, we have decided for femoral approach as no additional antiseptic skin preparation at the neck was needed.

In conclusion, we presented a case of a giant high-flow renal AVF successfully treated with coil embolization. Coil mass dislodgement was encountered during attempted transarterial double-catheter approach; transvenous approach allowed coil mass retrieval, an additional benefit of simultaneous pulmonary circulatory protection, and last but not least, successful reattempt of coil embolization of the fistula.

## Learning points

1. Different types of AVFs in terms of their angio-architecture which guide the choice of embolization material.
2. Comparing different vascular accesses to the renal AVFs (transarterial, transvenous and simultaneous transarterial and transvenous approaches).
3. The pitfall of distal coil migration in the commonly used transarterial approach of AVF access, especially in patients with giant high flow AVFs.
4. Transvenous approach allow simultaneous pulmonary circulatory protection and embolization of the fistula.
